# Exploring Relationships Between Tweet Numbers and Over-the-counter Drug Sales for Allergic Rhinitis: Retrospective Analysis

**DOI:** 10.2196/33941

**Published:** 2022-02-02

**Authors:** Shoko Wakamiya, Osamu Morimoto, Katsuhiro Omichi, Hideyuki Hara, Ichiro Kawase, Ryuji Koshiba, Eiji Aramaki

**Affiliations:** 1 Graduate School of Science and Technology Nara Institute of Science and Technology Nara Japan; 2 Scientific Affairs SSP Co Ltd Tokyo Japan; 3 Medical Affairs Sanofi KK Tokyo Japan

**Keywords:** infoveillance, social media, Twitter, over-the-counter drugs, allergic rhinitis, hay fever, drug, treatment, allergy, immunology, surveillance, monitoring, prevalence, motivation, Japan, symptom

## Abstract

**Background:**

Health-related social media data are increasingly being used in disease surveillance studies. In particular, surveillance of infectious diseases such as influenza has demonstrated high correlations between the number of social media posts mentioning the disease and the number of patients who went to the hospital and were diagnosed with the disease. However, the prevalence of some diseases, such as allergic rhinitis, cannot be estimated based on the number of patients alone. Specifically, individuals with allergic rhinitis typically self-medicate by taking over-the-counter (OTC) medications without going to the hospital. Although allergic rhinitis is not a life-threatening disease, it represents a major social problem because it reduces people’s quality of life, making it essential to understand its prevalence and people’s motives for self-medication behavior.

**Objective:**

This study aims to explore the relationship between the number of social media posts mentioning the main symptoms of allergic rhinitis and the sales volume of OTC rhinitis medications in Japan.

**Methods:**

We collected tweets over 4 years (from 2017 to 2020) that included keywords corresponding to the main nasal symptoms of allergic rhinitis: “sneezing,” “runny nose,” and “stuffy nose.” We also obtained the sales volume of OTC drugs, including oral medications and nasal sprays, for the same period. We then calculated the Pearson correlation coefficient between time series data on the number of tweets per week and time series data on the sales volume of OTC drugs per week.

**Results:**

The results showed a much higher correlation (*r*=0.8432) between the time series data on the number of tweets mentioning “stuffy nose” and the time series data on the sales volume of nasal sprays than for the other two symptoms. There was also a high correlation (*r*=0.9317) between the seasonal components of these time series data.

**Conclusions:**

We investigated the relationships between social media data and behavioral patterns, such as OTC drug sales volume. Exploring these relationships can help us understand the prevalence of allergic rhinitis and the motives for self-care treatment using social media data, which would be useful as a marketing indicator to reduce the number of out-of-stocks in stores, provide (sell) rhinitis medicines to consumers in a stable manner, and reduce the loss of sales opportunities. In the future, in-depth investigations are required to estimate sales volume using social media data, and future research could investigate other diseases and countries.

## Introduction

Social media data are a valuable source for rapidly exploring and understanding various real-world phenomena. Because many people share their health conditions on social media, a high volume of health-related social media data are available and the use of these data for large-scale quantitative analyses [1,2] and disease surveillance (referred to as “infoveillance”) is gaining much interest [3]. In particular, major advances have been made in the use of social media data to track the prevalence and spread of infectious diseases, including influenza and other conditions [4-13]. These studies have contributed to public health by demonstrating high correlations between fluctuations in the number of relevant social media posts and patients for a specific disease.

This study focuses on allergic rhinitis, also called hay fever, which is one of the most common allergic diseases worldwide [14]. In Japan, many people suffer from seasonal allergic rhinitis induced by Japanese cedar pollen between February and April each year. According to the results of the Japan National Epidemiological Studies in 1998, 2008, and 2019, the prevalence of allergic rhinitis in Japan has increased significantly over the past 20 years (49.2% increase in overall allergic rhinitis and 42.5% increase in cedar pollen–induced allergic rhinitis). It is now a national disease that affects the majority of the population [15].

Although allergic rhinitis is not a life-threatening disease, its main symptoms—sneezing, nasal discharge (watery), and nasal obstruction—significantly impair the quality of life (QOL) of patients, causing a major social problem [15-17]. According to the practical guideline for the management of allergic rhinitis in Japan 2020 [18], approximately 40% of people in Japan said they had allergic rhinitis, of which 30% had nasal allergies caused by pollen. In addition, since most self-medication for allergic rhinitis in Japan is for seasonal allergic rhinitis caused by cedar and cypress tree pollen, which are dispersed in the spring, the number of patients and the sales amount of over-the-counter (OTC) rhinitis drugs are greatly affected by the pollen conditions of the year or region. About three-fourths of OTC rhinitis drugs are oral medicines and one-fourth are nasal sprays; for oral medicines, more than 60% of annual sales are concentrated in the 3 main pollen dispersal months, from February to April. Retailers are required to assess the situation and make accurate predictions during the annual spring pollen season, as they are required to manage products without causing opportunity loss. Therefore, a real-time understanding of the prevalence of allergic rhinitis is important for providing necessary solutions, such as appropriate pharmaceutical distribution.

The surveillance methods currently available rely on the following three types of statistical data:

1. Amount of pollen. This value is not precise because of the complex mechanisms that cause allergic rhinitis. First, when antigens such as pollen or house dust enter the nose, sneezing and nasal discharge occur immediately. As antigens repeatedly enter the nose, a reaction centered on nasal congestion occurs, and the symptoms of allergic rhinitis intensify [15]. Thus, there is a time lag between exposure to pollen and the onset of allergic rhinitis. In addition, the timing of symptom onset varies from person to person. For some patients, symptoms appear as soon as pollen starts to disperse, while for others, symptoms do not appear until there is a large amount of pollen in the air. The intensity of symptoms is also not the same, with some people having mild symptoms and others having severe symptoms [15]. As a result, there is no strong association between pollen count and patient numbers, complicating disease surveillance.
2. Number of outpatients. This is the number of patients who visit the hospital for allergic rhinitis. Since many patients try to self-medicate using OTC drugs instead of visiting a medical institution, such data do not provide an overall picture of the trend.
3. Volume of OTC drug sales. This value would be more reliable than the above two types of data. The trend of self-medicating using OTC drugs is being accelerated by the introduction of many new OTC medicines that switched from prescription to OTC status [19].

So far, correlations of hay fever–related tweets with pollen counts [12] and reported incidents of hay fever [13] have been investigated. Our previous study [20] analyzed data on pollen count, the number of hay fever–related tweets, and the number of patients during the seasonal allergic rhinitis period in Japan to explore their relationships. The results showed that increased pollen counts were associated with increased numbers of tweets and patients. In addition, increases in the number of tweets were also associated with increased numbers of patients.

This study explores the relationships between social media data related to allergic rhinitis and OTC allergic rhinitis drug sales as an outcome of consumer behavior. To the best of our knowledge, this is the first study to compare tweet trends with drug sales trends. Specifically, we investigate the correlation between the weekly number of tweets related to 3 main symptoms of allergic rhinitis—paroxysmal repetitive sneezing (sneezing), watery rhinorrhea (runny nose), and nasal obstruction (stuffy nose)—and the weekly sales volume of OTC allergic rhinitis medication (oral medicine and nasal spray).

## Methods

### Data

#### Number of Tweets Related to Allergic Rhinitis Nasal Symptoms

We collected tweets that included any of the following Japanese keywords for major nasal symptoms of allergic rhinitis: kushami (くしゃみ; sneezing), hanamizu (鼻水; runny nose), and hanadumari (鼻づまり; stuffy nose). These keywords were selected by analyzing co-occurrence words in tweets concerning hay fever (kafunsho or 花粉症 in Japanese) and extracting typical notations with high frequency in our preliminary experiments. These tweets were crawled using the Twitter application programming interface. After removing retweets, we obtained 5,834,920 tweets concerning sneezing, 7,695,598 tweets concerning runny nose, and 274,119 tweets related to stuffy nose between January 2, 2017, and January 3, 2021 (209 weeks).

#### OTC Allergic Rhinitis Medication Sales Volume

In addition to visiting a medical institution for the treatment of allergic rhinitis, patients self-medicate using OTC drugs based on their own judgment. OTC allergic rhinitis medications are typically oral medicines or nasal sprays. In recent years, many medicines have switched from prescription to OTC status. Allergic rhinitis is one of the health complaints for which self-medication is most common.

Since there are no comprehensive government statistics or other survey information on the number of users of OTC drugs, estimates are made using sales data for OTC drugs. However, the reporting of OTC sales information also involves some delays. In addition to information on the shipment value of OTC drugs from manufacturers and distributors, point-of-sale (POS) data from retail stores (such as supermarkets, convenience stores, home centers/discount stores, drugstores, and pharmacies) provided by private research companies are used. POS data are collected almost in real time from approximately 6000 panel retailers nationwide through in-store cash registers and systems, and include information such as which products are sold, when, where, at what price, and how many; these data are provided after aggregation and are an important source of information for understanding consumer behavior regarding self-medication [21]. 

For this study, we used data on the sales volume of OTC allergic rhinitis drugs; the data were obtained from INTAGE Healthcare Inc’s nationwide drugstore panel research [19]. During the study period between January 2, 2017, and January 3, 2021, a total of 205 oral medicines (OTC allergic rhinitis drugs) recorded a weekly market share of 0.0001% or more (including Alesion 20 by SSP Co Ltd and Allegra FX by Hisamitsu Pharmaceutical Co Inc), as did 118 nasal spray products (such as Pabron Nasal Spray by Taisho Pharmaceutical Co Ltd and Contac Rhinitis Spray for seasonal allergies by GlaxoSmithKline plc). No new product launched after January 2017 had a weekly market share of more than 10% [21]. Therefore, we consider that new products have not had a significant impact on sales.

### Correlation Coefficient Calculation

We aimed to examine the relationships between allergic rhinitis nasal symptom–related tweet numbers and OTC allergic rhinitis medication sales volume (oral medicine or nasal spray). To this end, we calculated the Pearson correlation coefficients between the time series data. In addition to the correlations between the observed time series data, we also investigated correlations between the trend, seasonality, and residual components of these time series data. The time series decomposition was performed using the seasonal_decompose function from the statsmodels module [22] in Python.

## Results

**Figure 1:**
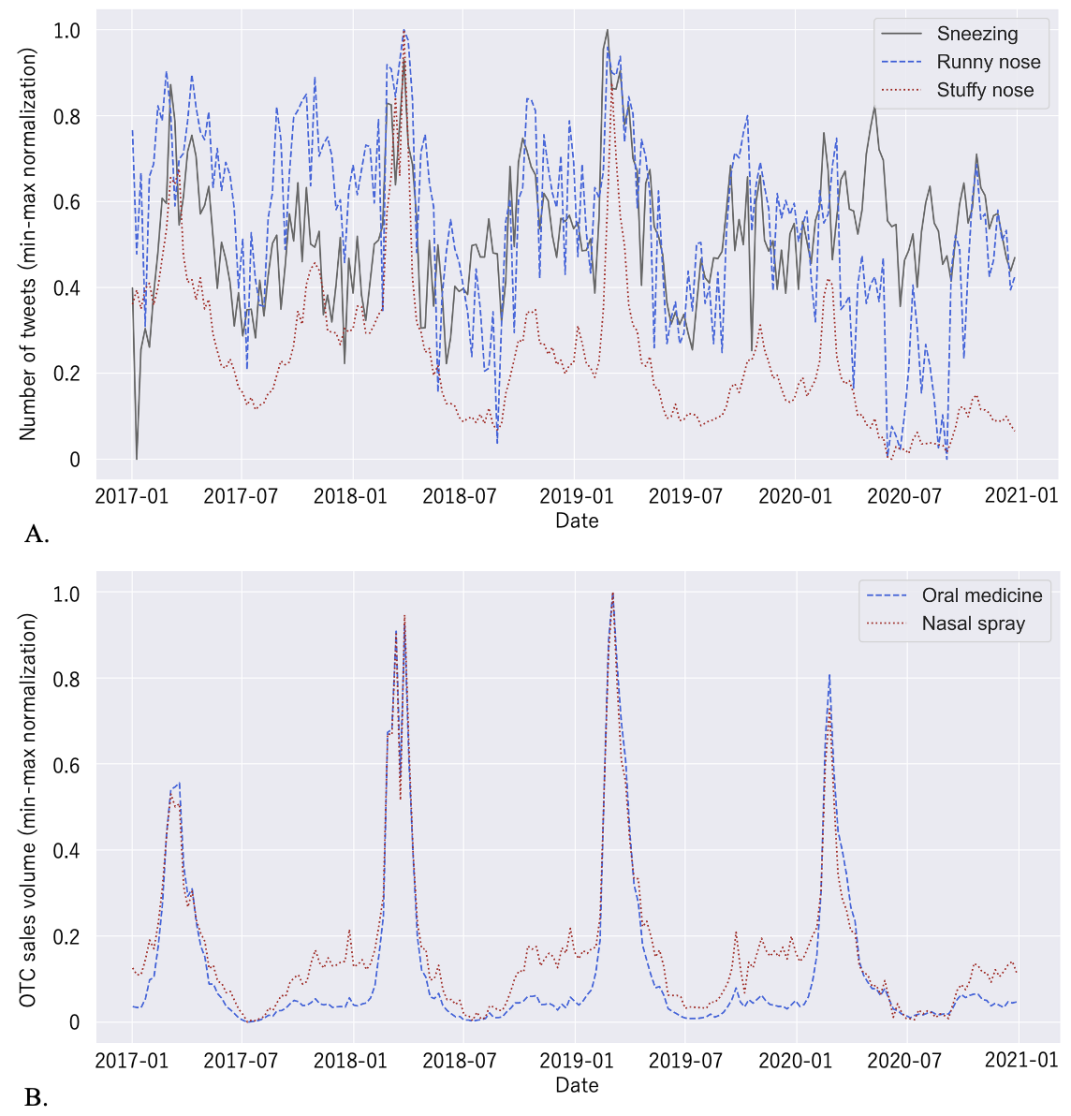
Time-based changes in data from 2017 to 2020 in Japan. The x-axes represent the date and the y-axes represent data counts, to which min-max normalization is applied for the following variables: (A) weekly changes in the number of tweets for the 3 main symptoms of allergic rhinitis (sneezing, solid gray line; runny nose, blue dashed line; and stuffy nose, red dotted line), and (B) weekly changes in the sales volume of OTC allergic rhinitis medication (oral medicine, blue dashed line; nasal spray, red dotted line). OTC: over-the-counter.

Figure 1A shows the changes in the weekly number of tweets related to the 3 main symptoms of allergic rhinitis for the target period. Figure 1B shows the changes in weekly sales volume of OTC allergic rhinitis medication, including oral medication and nasal spray, from 2017 to 2020. The most common causative antigen of seasonal allergic rhinitis in Japan is cedar pollen, which disperses between February and April. In Figure 1B, there is a clear peak during this period each year.

**Figure 2:**
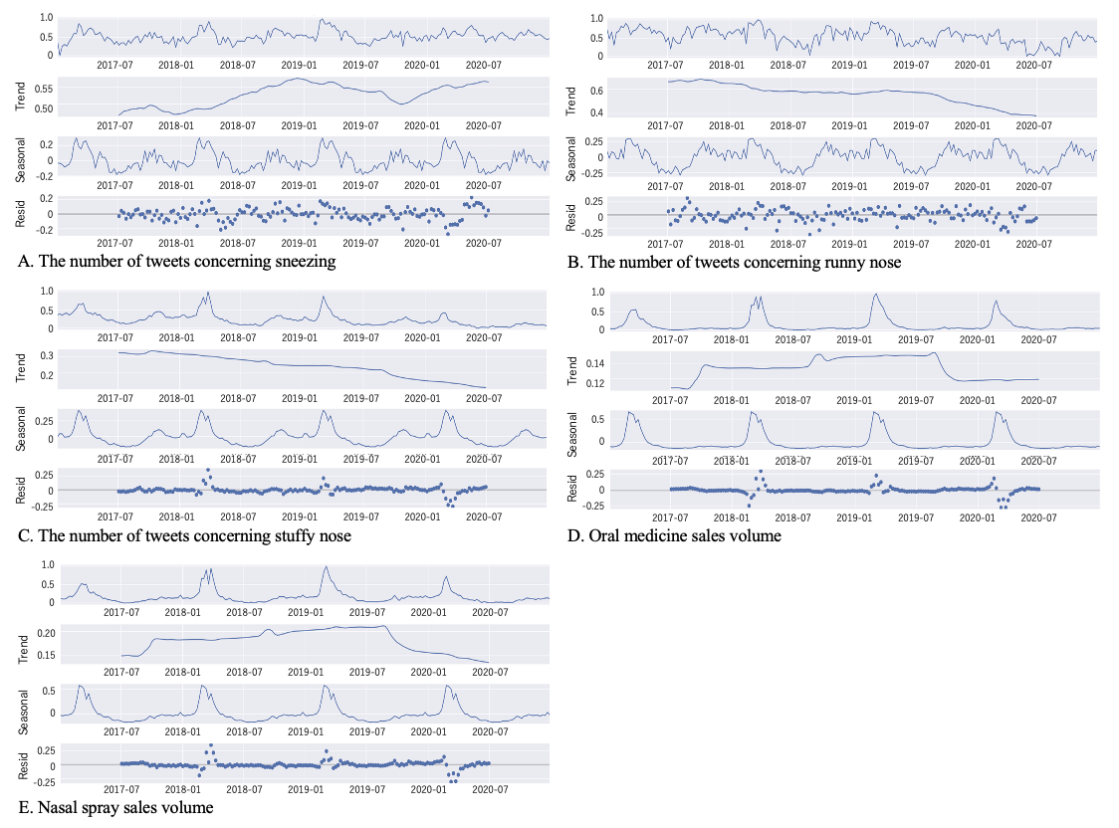
Time series of observed data (the top graph) and its decomposed components (the second, third, and fourth graphs represent trend, seasonality, and residual components, respectively) from 2017 to 2020. (A) Time series of the number of tweets concerning sneezing, (B) time series of the number of tweets concerning runny nose, (C) time series of the number of tweets concerning stuffy nose, (D) time series of oral medicine sales volume, and (E) time series of nasal spray sales volume. Resid: residual.

**Figure 3:**
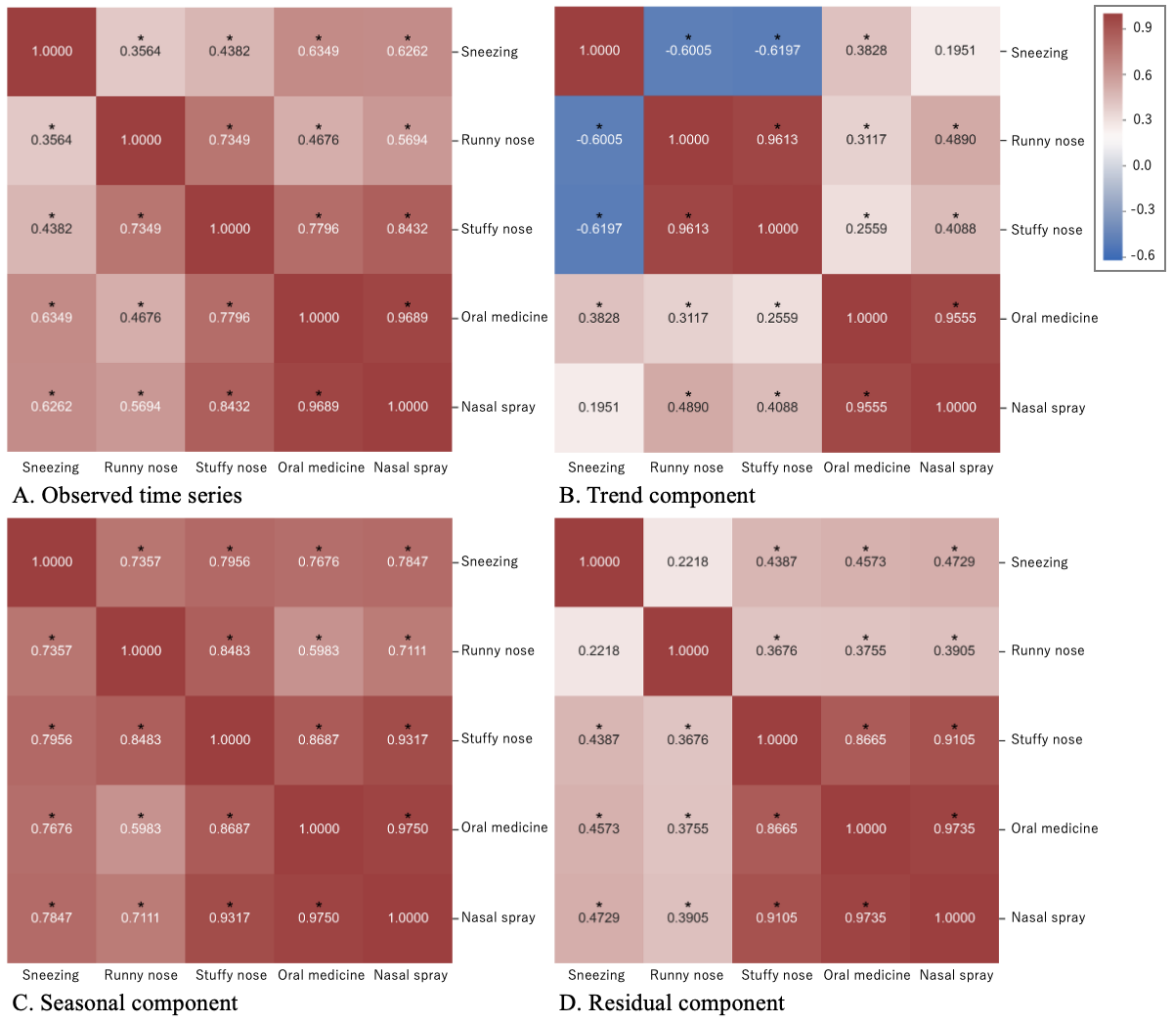
Heat map of time series correlation. (A) Correlations between observed time series data, (B) correlations between the trend components, (C) correlations between the seasonality components, and (D) correlations between the residual components. The trend, seasonality, and residual components were estimated by decomposing the observed time series data. *P<.001.

Figure 2 shows the time series of the observed data and its decomposed components: trend, seasonality, and residual. Figure 3 shows heat maps of correlations of all pairs of time series data. Figure 3A shows correlations between observed time series data. Figures 3B-D show correlations between the trend, seasonality, and residual components, respectively.

Among pairs of the observed time series of tweets, the positive correlation between tweets concerning stuffy nose and tweets concerning runny nose was the highest (*r*=0.7349), as shown in Figure 3A. The time series of the trend components showed the highest positive correlation (*r*=0.9613; Figure 3B), and the time series of the seasonal components was also highly correlated (*r*=0.8483; Figure 3C). On the other hand, there were positive correlations with tweets about sneezing, but they were not high (*r*=0.3564 for tweets concerning runny nose and *r*=0.4382 for tweets concerning stuffy nose; Figure 3A), due to negative correlations between the time series of the trend components (*r*=–0.6005 for tweets concerning runny nose and *r*=–0.6197 for tweets concerning stuffy nose; Figure 3B). As for OTC medication sales volume, the highest positive correlation (*r*>0.95) was between oral medicine and nasal spray.

As for the correlations between tweets and OTC medication sales volume, the highest positive correlation (*r*=0.8432) was between the time series of tweets concerning stuffy nose and the time series of nose spray sales volume, as shown in Figure 3A. The correlation of the trend component was positive and not high (*r*=0.4088; Figure 3B). On the other hand, the correlation of the seasonal component was positive and high (*r*=0.9317; Figure 3C), indicating the tweet numbers of allergic rhinitis keywords and OTC drug sales have a seasonal pattern due to the seasonality of allergic rhinitis in Japan. Several Twitter-based surveillance studies [7-11] dealt with infectious diseases that demonstrate seasonality, such as influenza, and some of them effectively utilized such seasonal features.

## Discussion

### Principal Results

We found that the positive correlation between the number of tweets concerning stuffy nose and the weekly sales volume of nasal spray is the highest. In various surveys, it has been reported that among the 3 main symptoms of allergic rhinitis, nasal congestion is the most unpleasant and difficult to cure and it reduces patients’ QOL, especially their mental QOL [15]. Therefore, it can be inferred that the inability to seek medical attention or the desire to deal with the symptoms immediately may appear stronger when people are experiencing nasal obstruction than sneezing or runny nose, which may lead to the purchasing of OTC allergic rhinitis medications. In particular, patients with nasal obstruction symptoms often use OTC nasal drops, which can be administered directly to the affected area (nasal cavity) in the hope that these will provide immediate relief, and a high correlation was observed in this study between tweets about nasal obstruction and sales volume of nasal sprays.

Sneezing and runny nose are symptoms that are obvious to people other than the patient and may also be tweeted about by other people, not just patients, which can create noise. Compared to these visible symptoms, symptoms such as nasal obstruction are less likely to be noticed by others and are often tweeted about by patients themselves, suggesting that tweets related to a stuffy nose create less noise. The numbers of tweets about sneezing and runny nose are much larger than the number of tweets about stuffy nose. Thus, when dealing with tweets about symptoms that are easily observable by others, it would be reasonable to distinguish between tweets by patients and those by others using natural language processing.

Furthermore, according to a survey in Japan [21], an increasing number of people are becoming sensitive to people sneezing near them, which spreads droplets, due to the COVID-19 pandemic. This may be one of the reasons why the time series of tweets concerning sneezing has a different trend than tweets about the other two symptoms, as shown in Figures 2A-C and Figure 3B.

### Limitations

This study applied simple statistical methods to explore the relationship between the number of social media posts mentioning the main symptoms of allergic rhinitis and the sales volume of OTC rhinitis medications in Japan. In the future, we need to further explore relations between the variables, including causal, temporal, and confounding relations.

Although this study focused on nasal symptoms of allergic rhinitis and investigated their relationship with OTC drug sales volume, there are other symptoms associated with allergic rhinitis, including sleep disturbance, olfactory disturbance, nasal itching, problems with learning, poor concentration, inattention, fatigue, irritability, lightheadedness, and headache. In the future, these symptoms should be considered when conducting further investigations. In addition, this study dealt with the sales amount of OTC drugs as the sales volume; in the future, we should consider the number of sales as well.

Another limitation is language bias. This paper focuses only on the Japanese language, in which several allergy symptoms are not polysemic. However, allergy symptoms in other languages may have multiple meanings. In addition to polysemic words, idioms, including allergy symptoms, could bias word frequencies. This aspect is worth studying in the future.

However, the actual number of patients with allergic rhinitis is still unknown. This study revealed only a correlation between the sales volume of OTC drugs and the number of tweets about the main symptoms of allergic rhinitis; the relationship of the sales volume with the number of patients will require further investigation.

### Conclusions

This study investigated correlations between social media data related to allergic rhinitis symptoms and OTC allergic rhinitis drug sales volume as an outcome of consumer behavior. We analyzed time series data for 4 years and showed a strong positive correlation between the number of tweets regarding stuffy nose and the sales volume of nasal spray. Regardless of the temporal dependency direction between the two variables, understanding such relationships has great potential as a market indicator to reduce the number of out-of-stocks in stores, provide (sell) rhinitis medicines to consumers in a stable manner, and reduce the loss of sales opportunities. In the future, additional relationships, such as causal, temporal, and confounding relations, should be explored by employing sophisticated time series analysis methods. In-depth investigations are also required to make estimations of sales volume using social media data, and future research could investigate other diseases and countries.

## Abbreviations

OTC: over-the-counter
POS: point-of-sale
QOL: quality of life

## References

[1] Lee JL, DeCamp M, Dredze M, Chisolm MS, Berger ZD (2014). What are health-related users tweeting? A qualitative content analysis of health-related users and their messages on twitter. J Med Internet Res.

[2] Alnemer KA, Alhuzaim WM, Alnemer AA, Alharbi BB, Bawazir AS, Barayyan OR, Balaraj FK (2015). Are Health-Related Tweets Evidence Based? Review and Analysis of Health-Related Tweets on Twitter. J Med Internet Res.

[3] Eysenbach G (2009). Infodemiology and infoveillance: framework for an emerging set of public health informatics methods to analyze search, communication and publication behavior on the Internet. J Med Internet Res.

[4] Charles-Smith LE, Reynolds TL, Cameron MA, Conway M, Lau EHY, Olsen JM, Pavlin JA, Shigematsu M, Streichert LC, Suda KJ, Corley CD (2015). Using Social Media for Actionable Disease Surveillance and Outbreak Management: A Systematic Literature Review. PLoS One.

[5] Paul M, Sarker A, Brownstein J, Nikfarjam A, Scotch M, Smith K (2016). Social media mining for public health monitoring and surveillance.

[6] Al-Garadi MA, Khan MS, Varathan KD, Mujtaba G, Al-Kabsi AM (2016). Using online social networks to track a pandemic: A systematic review. J Biomed Inform.

[7] Aramaki E, Maskawa S, Morita M (2011). Twitter catches the flu: Detecting influenza epidemics using Twitter. Proceedings of the 2011 Conference on Empirical Methods in Natural Language Processing.

[8] Paul M, Dredze M (2011). You are what you tweet: Analyzing Twitter for public health. Proceedings of the International AAAI Conference on Web and Social Media.

[9] Broniatowski DA, Paul MJ, Dredze M (2013). National and local influenza surveillance through Twitter: an analysis of the 2012-2013 influenza epidemic. PLoS One.

[10] Paul MJ, Dredze M, Broniatowski D (2014). Twitter improves influenza forecasting. PLoS Curr.

[11] Wakamiya S, Kawai Y, Aramaki E (2018). Twitter-Based Influenza Detection After Flu Peak via Tweets With Indirect Information: Text Mining Study. JMIR Public Health Surveill.

[12] Gesualdo F, Stilo G, D'Ambrosio A, Carloni E, Pandolfi E, Velardi P, Fiocchi A, Tozzi AE (2015). Can Twitter Be a Source of Information on Allergy? Correlation of Pollen Counts with Tweets Reporting Symptoms of Allergic Rhinoconjunctivitis and Names of Antihistamine Drugs. PLoS One.

[13] Quincey E, Kyriacou T, Pantin T (2016). #hayfever; A longitudinal study into hay fever related tweets in the UK. Proceedings of the 6th International Conference on Digital Health Conference.

[14] Bousquet J, Anto JM, Bachert C, Baiardini I, Bosnic-Anticevich S, Walter Canonica G, Melén Erik, Palomares Oscar, Scadding Glenis K, Togias Alkis, Toppila-Salmi Sanna (2020). Allergic rhinitis. Nat Rev Dis Primers.

[15] Okubo K, Kurono Y, Ichimura K, Enomoto T, Okamoto Y, Kawauchi H, Suzaki H, Fujieda S, Masuyama K, Japanese Society of Allergology (2020). Japanese guidelines for allergic rhinitis 2020. Allergol Int.

[16] Yamada T, Saito H, Fujieda S (2014). Present state of Japanese cedar pollinosis: the national affliction. J Allergy Clin Immunol.

[17] Oseroff C, Pham J, Frazier A, Hinz D, Sidney J, Paul S, Greenbaum JA, Vita R, Peters B, Schulten V, Sette A (2016). Immunodominance in allergic T-cell reactivity to Japanese cedar in different geographic cohorts. Ann Allergy Asthma Immunol.

[18] Okano M (2021). Practical guideline for the management of allergic rhinitis in Japan 2020. Arerugi.

[19] SRI+ (nationwide drugstore panel research). INTAGE Healthcare Inc.

[20] Wakamiya S, Matsune S, Okubo K, Aramaki E (2019). Causal Relationships Among Pollen Counts, Tweet Numbers, and Patient Numbers for Seasonal Allergic Rhinitis Surveillance: Retrospective Analysis. J Med Internet Res.

[21] Press release [in Japanese]. NOVARTIS.

[22] statsmodels v0.13.1.

